# Antiviral activity of silymarin and baicalein against dengue virus

**DOI:** 10.1038/s41598-021-98949-y

**Published:** 2021-10-27

**Authors:** Zhao Xuan Low, Brian Ming OuYong, Pouya Hassandarvish, Chit Laa Poh, Babu Ramanathan

**Affiliations:** 1 Department of Biological Sciences, School of Medical and Life Sciences, Sunway University, Kuala Lumpur, Malaysia; 2 Centre for Virus and Vaccine Research, School of Medical and Life Sciences, Sunway University, Kuala Lumpur, Malaysia; 3 Tropical Infectious Diseases Research and Education Centre, University Malaya, 50603 Kuala Lumpur, Malaysia

**Keywords:** Drug discovery, Microbiology, Diseases, Medical research

## Abstract

Dengue is an arthropod-borne viral disease that has become endemic and a global threat in many countries with no effective antiviral drug available currently. This study showed that flavonoids: silymarin and baicalein could inhibit the dengue virus in vitro and were well tolerated in Vero cells with a half-maximum cytotoxic concentration (CC_50_) of 749.70 µg/mL and 271.03 µg/mL, respectively. Silymarin and baicalein exerted virucidal effects against DENV-3, with a selective index (SI) of 10.87 and 21.34, respectively. Baicalein showed a better inhibition of intracellular DENV-3 progeny with a SI of 7.82 compared to silymarin. Baicalein effectively blocked DENV-3 attachment (95.59%) to the Vero cells, while silymarin prevented the viral entry (72.46%) into the cells, thus reducing viral infectivity. Both flavonoids showed promising antiviral activity against all four dengue serotypes. The in silico molecular docking showed that silymarin could bind to the viral envelope (E) protein with a binding affinity of − 8.5 kcal/mol and form hydrogen bonds with the amino acids GLN120, TRP229, ASN89, and THR223 of the E protein. Overall, this study showed that silymarin and baicalein exhibited potential anti-DENV activity and could serve as promising antiviral agents for further development against dengue infection.

## Introduction

Dengue is a mosquito-borne viral disease circulated mainly in the tropical and subtropical regions. In the past 50 years, dengue cases have increased 30 times and expanded to over 100 countries, both in urban and rural areas^1^. A total of 2.5 billion people worldwide are at risk, with a global annual infection of 390 million cases, including 500,000 hospitalizations and approximately 25,000 deaths^2^. Dengue is listed as one of the diseases that constitutes a public health emergency of international concern. Dengue infection is caused by the dengue virus (DENV), which belongs to the family *Flaviviridae* and genus *Flavivirus*. DENV is classified into four closely related serotypes, DENV-1, 2, 3, and 4, which share 65–70% sequence homology^3^. Dengue virus is an enveloped virus with a single-stranded RNA genome of approximately 11 kb^4^. Dengue infection has a broad spectrum of clinical presentations with unexpected clinical outcomes. With the rising concern of this disease, there is an urgent need for medical intervention to treat and prevent dengue infection. However, there is no universal vaccine or antiviral agent for the treatment of dengue infection. Dengvaxia (CYD-TDV) licensed in few dengue endemic countries by Sanofi Pasteur, a tetravalent vaccine with genes encoding the precursor membrane (prM) and envelope (E) proteins of DENV-1 to 4, inserted into the backbone of live attenuated yellow fever vaccine strain 17D^5^. However, the moderate to low efficacy of CYD-TDV against serotypes DENV-1 and 2 with limited protection of the seronegative vaccine recipients and those under 9 years old raised concerns over the safety and efficacy of this vaccine in some countries^6^. Intravenous fluid replacement therapy remains the only option that reduces the fatality rate below 1% in severe dengue cases.

Nature is an excellent reservoir of potent compounds that can directly be used as pharmaceuticals or compounds that can be developed into new drugs. Natural compounds derived from plants have been investigated as antiviral agents against DENV. For example, quercetin, naringenin, catechin, and chrysin are flavonoids extracted from plants and were reported to have anti-dengue activity^7–9^. Silymarin, a plant-derived flavonoid extracted from milk thistle, was reported to be a promising antiviral candidate against chikungunya virus (CHIKV) by reducing the viral replication and down-regulation of viral proteins, nsP1, nsP3, and E2E1^10^. Besides, silymarin was also reported to inhibit NS5B polymerase, suppress the TNF-α activation of NF-kB dependent transcription, and reduce the inflammation in hepatitis C virus (HCV) infection^11^. Recently, silymarin and baicalein were reported to be potent antiviral agents for Enterovirus 71 (EV-A71)^12^. Silymarin was safe during a clinical trial to evaluate its hepatoprotective properties in patients with liver cirrhosis^13^. Baicalein, a flavonoid isolated from the root of *Scutellaria baicalensis* and its derivative baicalin were reported to exhibit anti-DENV-2 activity in vitro^14,15^. A study also showed that baicalin exhibited antiviral activity against EV-71 by suppressing the Fas/FasL pathway and 3D polymerase^16^. The anti-dengue potential of silymarin is unknown, and the inhibitory effects of baicalein and baicalin on other dengue serotypes have not been reported, with the exception of DENV-2. Therefore, in this study, we aimed to determine the antiviral potential of silymarin, baicalein, and baicalin against DENV. Out of the three flavonoids investigated, silymarin and baicalein showed the potential to inhibit DENV-3, specifically by direct extracellular virucidal action. In addition, we showed that silymarin and baicalein significantly interfered with DENV-3 attachment and entry to Vero cells, respectively. To our knowledge, this is the first study demonstrating the antiviral effects of silymarin and baicalein against all four dengue serotypes in vitro.

## Results

### Cytotoxicity effects of flavonoids in Vero cells

**Figure 1:**
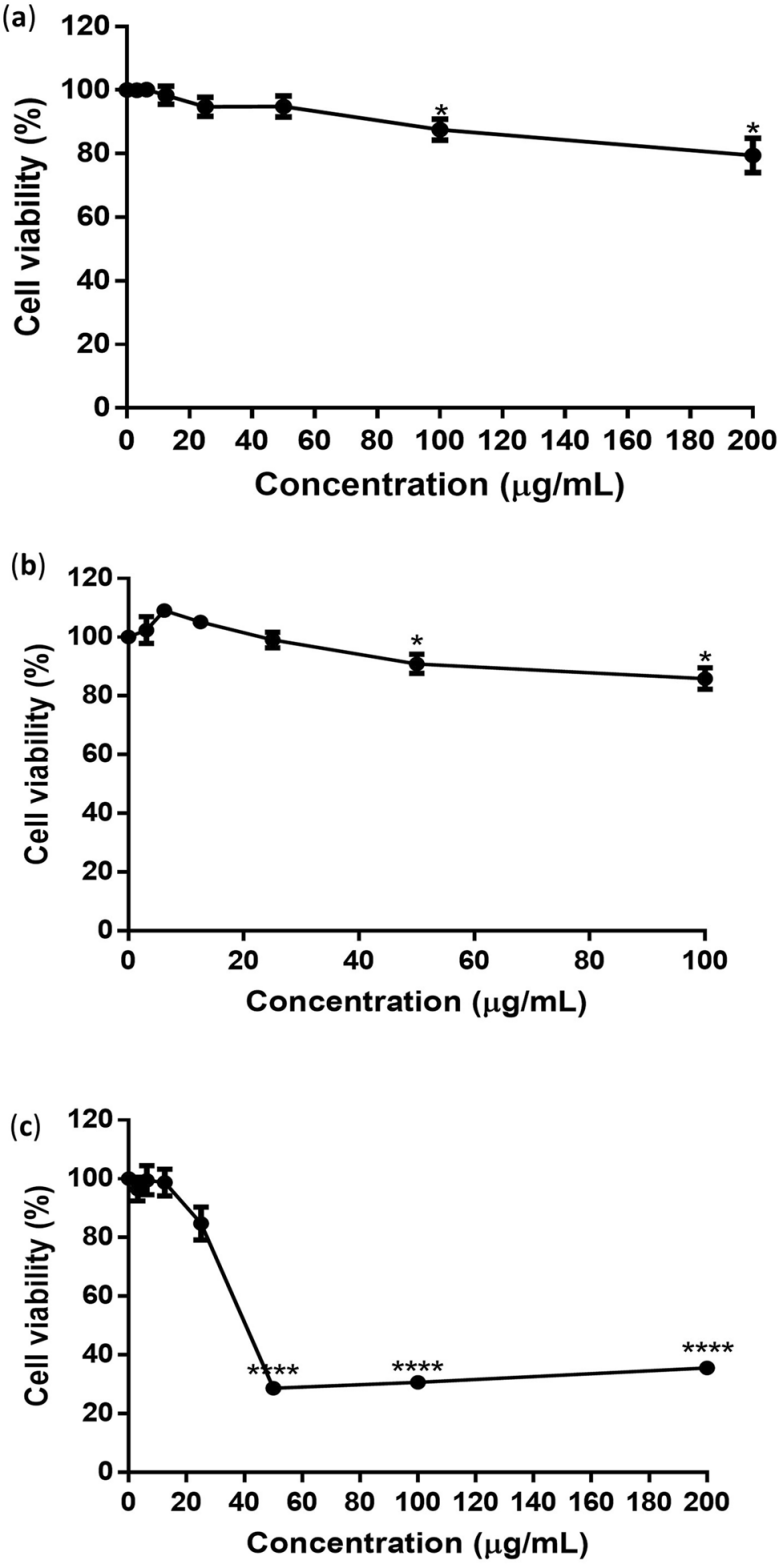
Cytotoxic effects of (**a**) silymarin, (**b**) baicalein, and (**c**) baicalin in Vero cells. Flavonoids were serially diluted in DMEM supplemented with 2% FBS, and Vero cells were treated with the diluted flavonoids for up to 48 h. The cytotoxicity of each flavonoid was determined by MTS assay using a microplate reader to measure the absorbance at 490 nm. Data presented as mean ± SEM. Error bars indicate the range of values obtained from three independent experiments. *P < 0.05, ****P < 0.0001 indicates a significant difference compared to the negative control analysed by the t-test.

The cytotoxicity effects of silymarin, baicalein, and baicalin in Vero cells were evaluated using MTS assay, and the CC_50_ and MNTD_80_ values were presented in Table 1. Concentrations up to 200 µg/mL for silymarin (Fig. 1a), 100 µg/mL for baicalein (Fig. 1b), and 20 µg/mL for baicalin (Fig. 1c) showed no significant cytotoxic effects in Vero cells after 48 h of incubation. The half-maximum cytotoxic concentration (CC_50_) of silymarin, baicalein, and baicalin was 749.70 ± 3.41 µg/mL, 271.03 ± 6.57 µg/mL, and 54.64 ± 0.76 µg/mL, respectively. The maximum-non-toxic-dose (MNTD_80_) of silymarin, baicalein and baicalin was 202.30 ± 3.68 µg/mL, 122.95 ± 2.37 µg/mL, and 19.15 ± 0.07 µg/mL respectively. Based on the MNTD_80_ values, 200 µg/mL of silymarin, 100 µg/mL of baicalein, and 20 µg/mL of baicalin showed more than 80% cell viability and were chosen as the highest concentrations evaluated in the antiviral assays.

**Table 1:** Cytotoxicity of flavonoids against Vero cells.

Compound	CC_50_ ± SEM (µg/mL)	MNTD_80_ ± SEM (µg/mL)
Silymarin	749.70 ± 3.41	202.30 ± 3.68
Baicalein	271.03 ± 6.57	122.95 ± 2.37
Baicalin	54.64 ± 0.76	19.15 ± 0.07

Different concentrations of silymarin, baicalein, and baicalin were tested for cytotoxicity in Vero cells by MTS assay. CC_50_ refers to the half maximum cytotoxic concentration, whereas MNTD80 is the dose at which 80% of the cells are viable. Data were acquired from three independent experiments and presented as mean ± standard error mean (SEM).

### Virucidal activity of silymarin and baicalein against DENV-3

**Figure 2:**
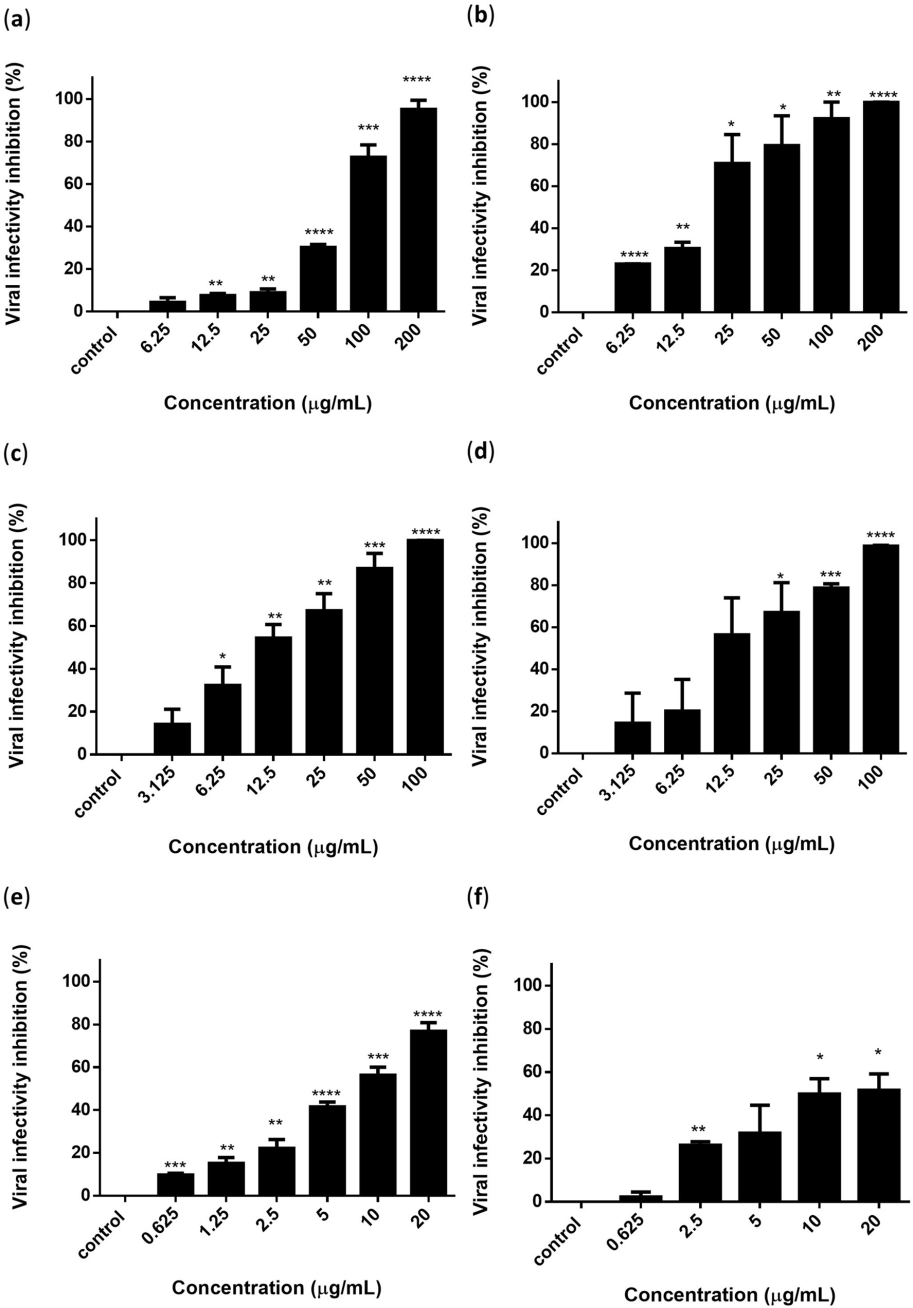
Silymarin, baicalein, and baicalin exhibited virucidal activity against extracellular DENV-3 in a dose-dependent manner. The inhibition of viral infectivity by silymarin was determined by (**a**) FFURA and (**b**) quantification of RNA copy number. Inhibition of viral infectivity by baicalein was determined by (**c**) FFURA and (**d**) quantification of RNA copy number. Inhibition of viral infectivity by baicalin was determined by (**e**) FFURA and (**f**) quantification of RNA copy number. Data presented as mean ± SEM. Error bars indicate the range of values obtained from three independent experiments. *P < 0.05, **P < 0.01, ***P < 0.001, ****P < 0.0001 indicates a significant difference compared to the negative control analyzed by the *t*-test.

Virucidal assays were performed to evaluate the viral inhibitory effects of flavonoids against DENV-3. Our findings showed that silymarin (Fig. 2a,b), baicalein (Fig. 2c,d), and baicalin (Fig. 2e,f) could exert direct dose-dependent virucidal effects against DENV-3. Based on the reduction in virus foci, the half-maximum inhibitory concentration (IC_50_) values of silymarin, baicalein, and baicalin were 68.94 ± 4.18 µg/mL, 12.70 ± 3.49 µg/mL, and 7.38 ± 1.10 µg/mL, respectively and the selective index (SI) values were 10.87, 21.34, and 7.40, respectively (Table 2). Silymarin achieved 95.13% of viral foci reduction at 200 µg/mL, baicalein achieved 99.78% of viral foci reduction at 100 µg/mL, and baicalin achieved 76% of viral foci reduction at 20 µg/mL. Based on the reduction in viral RNA copy number, the IC_50_ of silymarin, baicalein and baicalin were 18.48 ± 2.55 µg/mL, 17.24 ± 2.48 µg/mL, and 17.40 ± 6.51 µg/mL, respectively, and the SI values were 40.57, 15.72, and 3.14, respectively (Table 2). The SI values of all three flavonoids showed that these compounds are safe and effective against DENV-3.

**Table 2:** The half-maximum inhibitory concentration (IC_50_) and selective index (SI) of flavonoids against extracellular DENV-3 in virucidal assays.

Compound	CC_50_ (µg/mL)	IC_50_ (µg/mL)		SI	
		FFURA	qRT-PCR	FFURA	qRT-PCR
Silymarin	749.70 ± 30.41	68.94 ± 4.18	18.48 ± 2.55	10.87	40.57
Baicalein	271.03 ± 6.57	12.70 ± 3.49	17.24 ± 2.48	21.34	15.72
Baicalin	54.64 ± 0.76	7.38 ± 1.10	17.40 ± 6.51	7.40	3.14

IC50 refers to the 50% inhibitory concentration required to inhibit the virus and was obtained from virucidal assays. SI is a selective index (CC_50_/IC_50_). FFURA measures the infectious virus particles while qRT-PCR measures the viral RNA copy number. Data were acquired from three independent experiments and presented as mean ± standard error mean (SEM).

**Figure 3:**
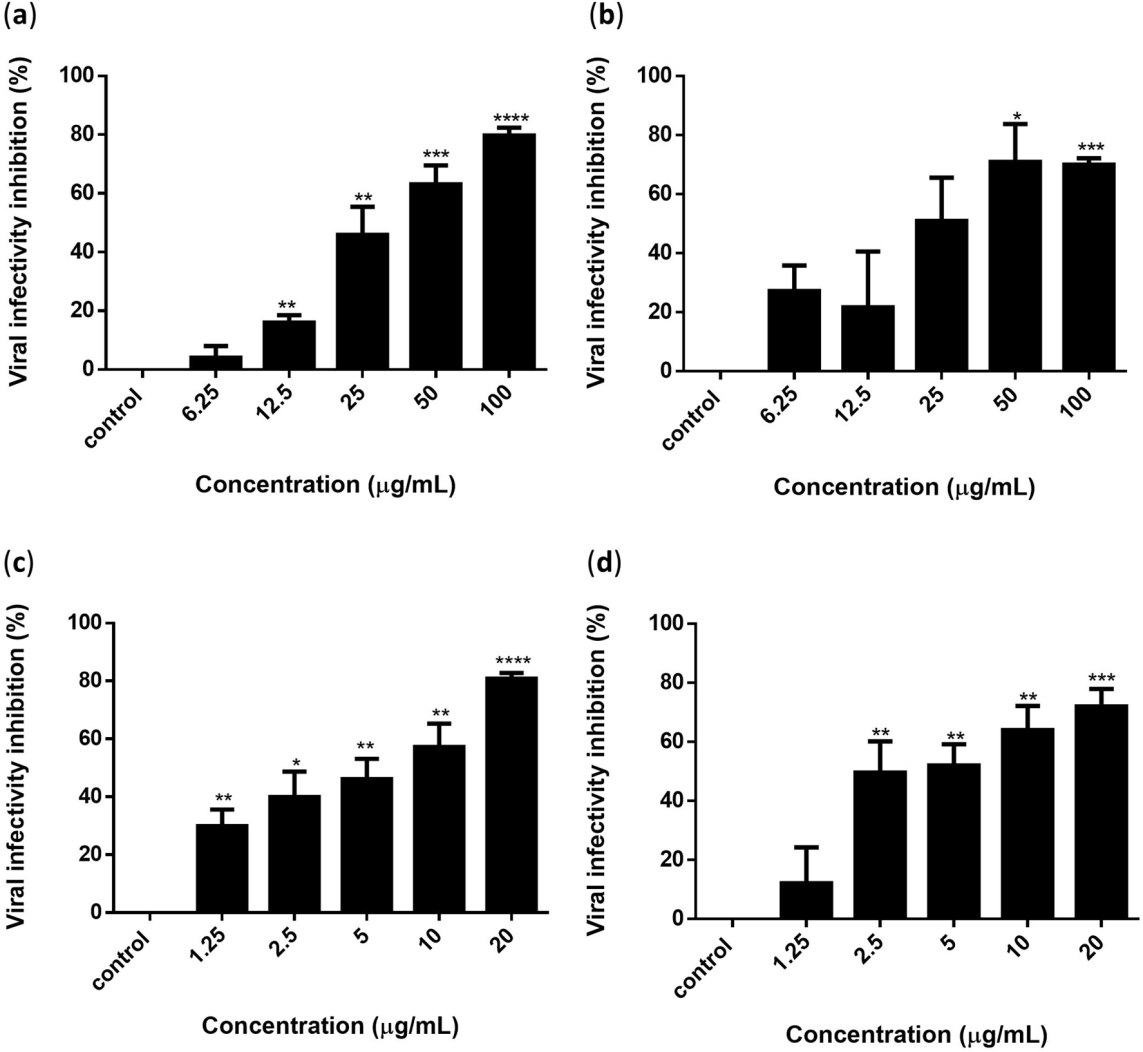
Baicalein and baicalin exhibited virucidal activity against DENV-3 progeny in a dose-dependent manner. Inhibition of viral progeny infectivity by baicalein was determined by (**a**) FFURA and (**b**) quantification of RNA copy number. Inhibition of viral progeny infectivity by baicalin was determined by (**c**) FFURA and (**d**) quantification of RNA copy number. Data presented as mean ± SEM. Error bars indicate the range of values obtained from three independent experiments. *P < 0.05, **P < 0.01, ***P < 0.001, ****P < 0.0001 indicates a significant difference compared to the negative control analyzed by the *t*-test.

To further evaluate the activity of flavonoids on inhibiting the intracellular viral progeny, Vero cells were infected with DENV-3, followed by treatment with silymarin, baicalein, and baicalin. After that, the viral progeny was collected and quantified using FFURA and qRT-PCR. The results showed that baicalein (Fig. 3a,b) and baicalin (Fig. 3c,d) could inhibit DENV-3 progeny in a dose-dependent manner. Based on the reduction in virus foci, the IC_50_ values of baicalein and baicalin were 34.66 ± 6.71 µg/mL and 5.31 ± 1.96 µg/mL, and the SI values were 7.82 and 10.29 respectively (Table 3). Baicalein (100 µg/mL) and baicalin (20 µg/mL) achieved maximum inhibition of 79.73% and 80.97% of intracellular viral progeny infectivity, respectively (Fig. 3). Based on the reduction in viral RNA copy number, the IC_50_ values of baicalein and baicalin were 29.70 ± 12.83 µg/mL and 4.53 ± 0.62 µg/mL, and the SI values were 9.13 and 12.06, respectively (Table 3). However, silymarin did not show any effect against intracellular DENV3 progeny infectivity, and all three flavonoids showed no cell protection activity when the treated cells were infected with DENV-3 (Supplementary Figure 2).

**Table 3:** The half-maximum inhibitory concentration (IC_50_) and selective index (SI) of flavonoids against DENV-3 progeny.

Compound	CC_50_ (µg/mL) ± SEM	IC_50_ (µg/mL) ± SEM		SI	
		FFURA	qRT-PCR	FFURA	qRT-PCR
Baicalein	271.03 ± 6.57	34.66 ± 6.71	29.70 ± 12.83	7.82	9.13
Baicalin	54.64 ± 0.76	5.31 ± 1.96	4.53 ± 0.62	10.29	12.06

IC_50_ refers to the 50% inhibitory concentration required to inhibit the viral progeny and was obtained from the post-infection assay. SI is a selective index (CC_50_/IC_50_). FFURA measures the infectious virus particles, while qRT-PCR measures the viral RNA copy number. Data were acquired from three independent experiments and presented as mean ± standard error mean (SEM).

### Baicalein required the shortest period of contact with DENV-3 to exert virucidal activity

**Figure 4:**
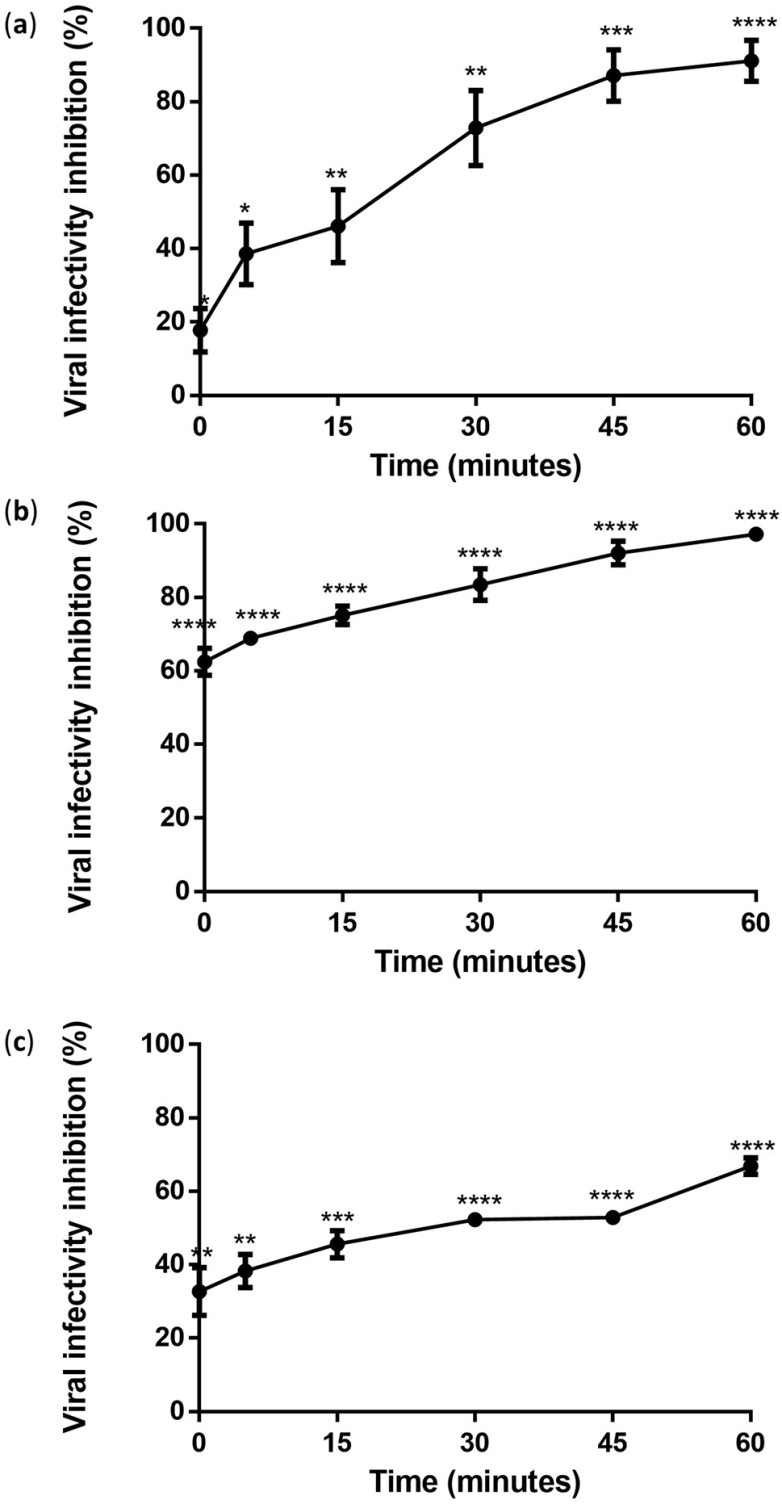
Extracellular virucidal activities of silymarin, baicalein, and baicalin against DENV-3 at different co-incubation times. (**a**–**c**) showed the inhibition of viral infectivity by silymarin (200 μg/mL), baicalein (100 μg/mL) and baicalin (20 μg/mL) after co-incubated with DENV-3 for 0, 5, 15, 30, 45 and 60 min at 37 °C. The flavonoid-treated DENV-3 from each time point was then used to infect Vero cells. Data presented as mean ± SEM. Error bars indicate the range of values obtained from three independent experiments. *P < 0.05, **P < 0.01, ***P < 0.001, ****P < 0.0001 indicates a significant difference compared to the negative control (0 μg/mL) analysed by *t*-test.

Time course assay was used to determine the effective time required for the three flavonoids to exhibit their virucidal effect against DENV-3. Flavonoids were treated with DENV-3 at different co-incubation times (0, 5, 15, 30, 45, and 60 min). Silymarin, baicalein, and baicalin required 17.4, 0, and 25.8 min co-incubation time to elicit 50% inhibition of viral infectivity, respectively (Table 4). Silymarin showed maximum inhibition of 90.12% when it was co-incubated with the virus for 60 min but only achieved a 17.8% virucidal effect when co-incubated with the virus for 0 min (Fig. 4a). On the other hand, baicalein showed a 62.45% virucidal effect at 0 min, and the inhibitory effects were progressively observed to be higher and maximum inhibition of 97.24% was achieved at 60 min (Fig. 4b). Baicalin showed a 32.69% virucidal effect at an incubation of 0 min and only achieved 66.9% inhibition even after 60 min of co-incubation with DENV-3 (Fig. 4c).

**Table 4:** The time taken to exert 50% viral infectivity inhibition of flavonoids against DENV-3.

Compound (concentration)	Time taken to achieve 50% viral infectivity inhibition (min)	Viral infectivity inhibition (%) achieved at 0 min ± SEM
Silymarin (200 µg/mL)	17.4	17.80 ± 5.9
Baicalein (100 µg/mL)	0	62.45 ± 3.67
Baicalin (20 µg/mL)	25.8	32.69 ± 6.48

Data were acquired from three independent experiments and presented as mean ± standard error mean (SEM).

### Flavonoids blocked the viral attachment and entry of DENV-3 into Vero cells

**Figure 5:**
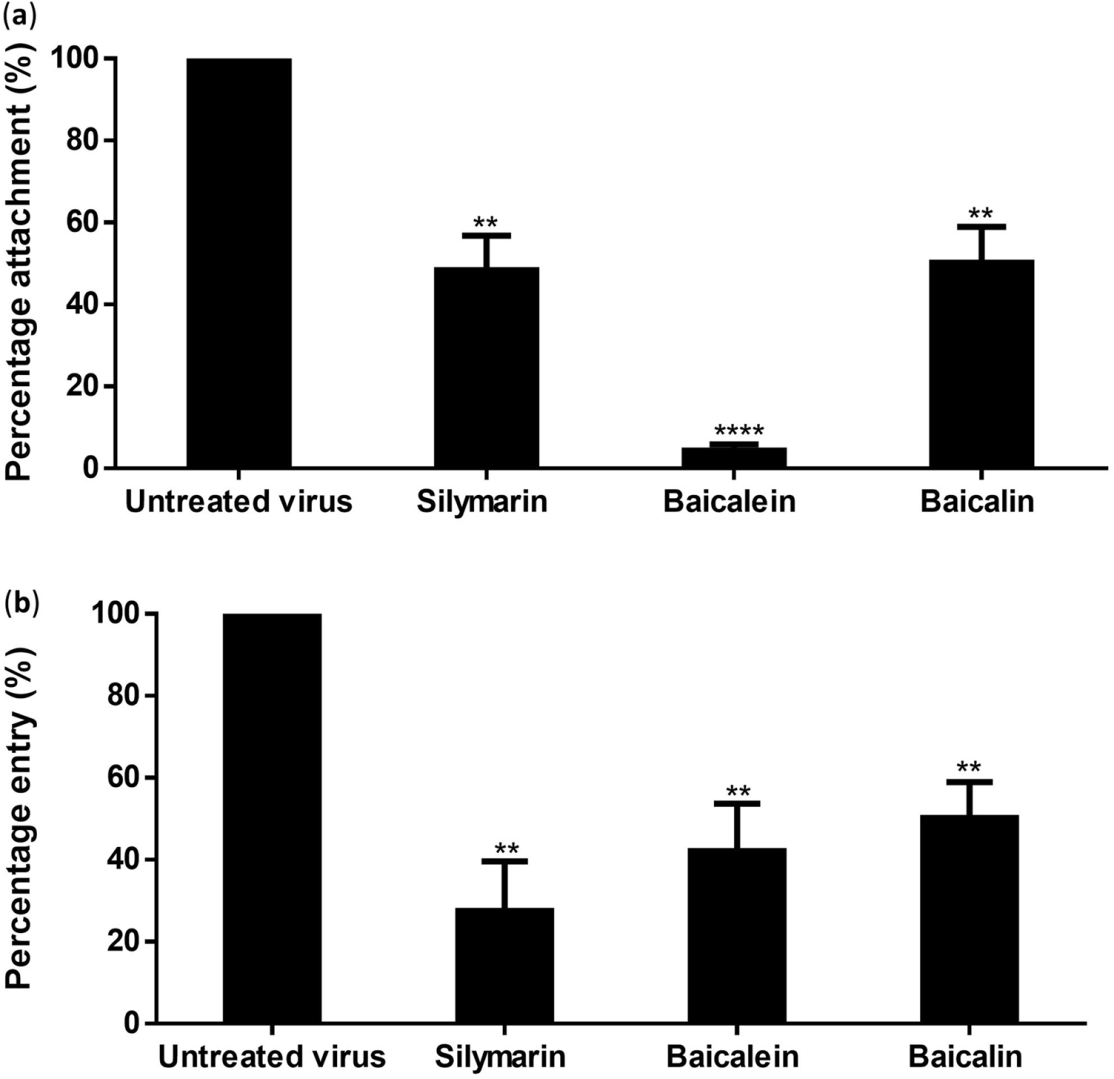
Effects of flavonoids on the attachment and entry of DENV-3 to Vero cells. The viral attachment (**a**) and entry (**b**) inhibition were calculated as the number of foci formed by the silymarin, baicalein, and baicalin-treated virus compared to the untreated virus. Data presented as mean ± SEM. Error bars indicate the range of values obtained from three independent experiments. **P < 0.01, ****P < 0.0001 indicates a significant difference between groups compared by *t*-test.

An attachment assay was carried out to evaluate the efficiency of flavonoids to block the viral attachment. The reduction in foci number showed that silymarin, baicalein, and baicalin at concentrations of 200 µg/mL, 100 µg/mL, and 20 µg/mL respectively were able to block 51.63%, 95.59%, and 48.52% of virus attachment to Vero cells (Fig. 5a, Table 5). Entry assay was carried out to evaluate the ability of the three flavonoids to inhibit viral internalization activity into Vero cells. The reduction in foci showed that silymarin, baicalein, and baicalin at concentrations of 200 µg/mL, 100 µg/mL, and 20 µg/mL respectively inhibited 72.46%, 57.91%, and 49.82% of viral entry (Fig. 5b, Table 5).

**Table 5:** Inhibition of DENV-3 viral attachment and entry by silymarin, baicalein, and baicalin.

Compound (concentration)	Inhibition of viral attachment (%)	Inhibition of viral entry (%)
Silymarin (200 µg/mL)	51.63 ± 8.46	72.46 ± 12.09
Baicalein (100 µg/mL)	95.59 ± 1.44	57.91 ± 11.65
Baicalin (20 µg/mL)	48.52 ± 9.10	49.82 ± 8.78

Data were acquired from three independent experiments and presented as mean ± standard error mean (SEM).

### FACS neutralization test (FNT)

**Figure 6:**
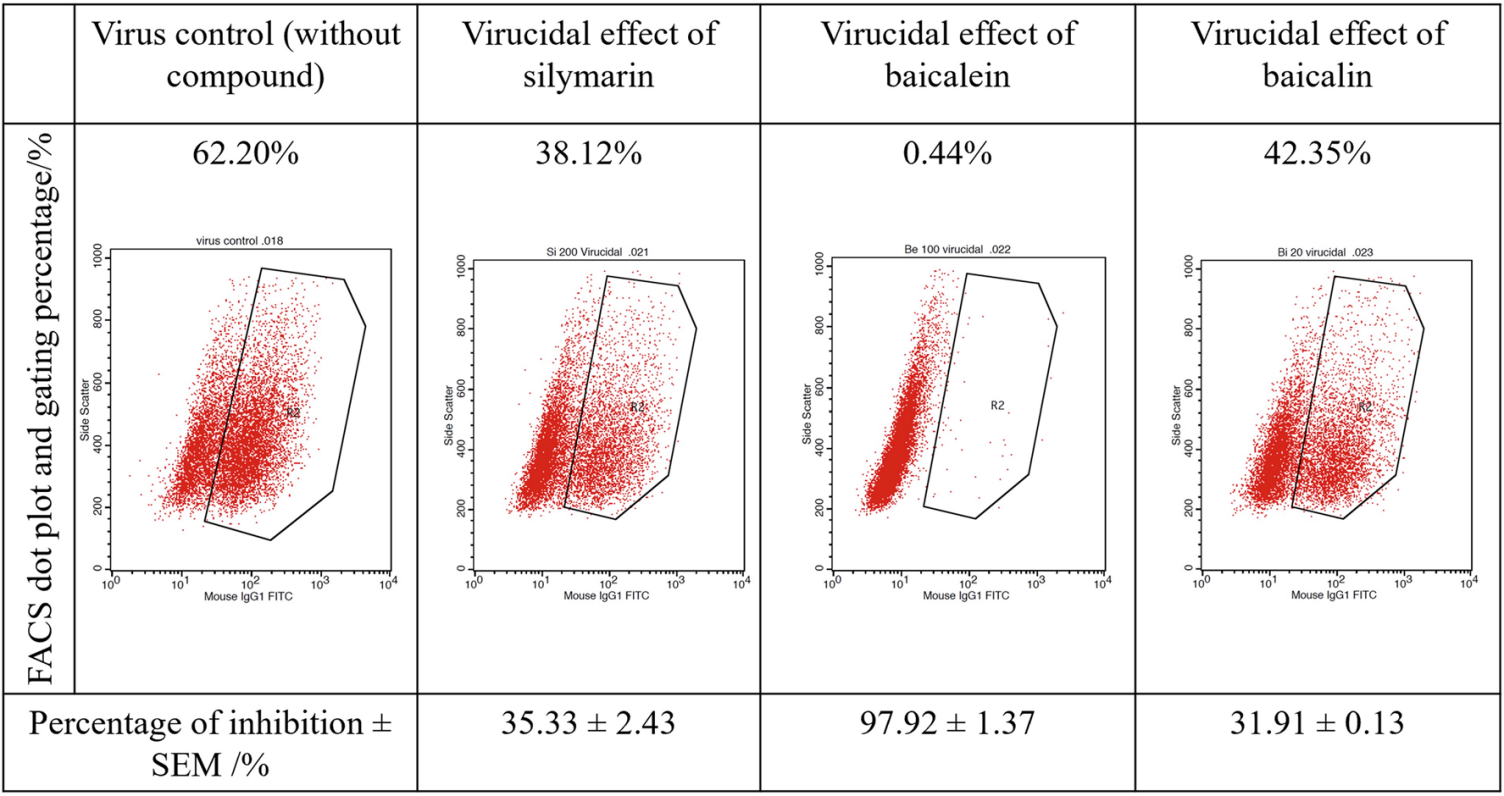
FACS-based assay to measure flavonoids' virucidal activity against DENV-3. For each sample, 10,000 events were collected and analyzed. Among the 10,000 events, the FITC-positive population for each sample was shown. The percentage of inhibition was calculated as the percentage of infected cells with flavonoid treatment compared to the percentage of infected cells without flavonoid treatment. Results were represented as the mean ± standard error of the mean (SEM) from triplicate assays determined from three independent experiments.

FACS assay was an alternative way to measure the ability of a compound to neutralize DENV. In the FNT, Vero cells were infected with DENV-3 at an MOI of 0.5. DENV-3 infected cells were detected using DENV-3 specific primary antibody and FITF-conjugated secondary antibody**.** The virucidal activity of flavonoids was evaluated in FNT, and the results showed that silymarin (200 µg/mL), baicalein (100 µg/mL), and baicalin (20 µg/mL) could neutralize 35.33%, 97.92%, and 31.91% of DENV-3 infected cell's population, respectively (Fig. 6).

### Silymarin and baicalein are able to reduce the infectivity of all four dengue serotypes

Silymarin and baicalein were tested against all four dengue serotypes to evaluate the broad-spectrum virucidal effects of these flavonoids through FFURA. Based on the reduction in foci number, silymarin (200 µg/mL) and baicalein (100 µg/mL) showed 97.20 ± 0.38% and 96.68 ± 2.10% inhibition of DENV-1; 76.94 ± 3.36% and 97.16 ± 1.41% inhibition of DENV-2; 99.39 ± 0.19% and 99.68 ± 0.10% inhibition of DENV-3; and 73.41 ± 2.18% and 80.49 ± 0.26% inhibition of DENV-4, respectively (Table 6).

**Table 6:** Virucidal activity of silymarin and baicalein against all four dengue serotypes.

Flavonoids (concentration)	Viral infectivity inhibition (%) ± SEM			
	DENV-1	DENV-2	DENV-3	DENV-4
Silymarin (200 µg/mL)	97.20 ± 0.38	76.94 ± 3.36	99.39 ± 0.19	73.41 ± 2.18
Baicalein (100 µg/mL)	96.68 ± 2.10	97.16 ± 1.41	99.68 ± 0.10	80.49 ± 0.26

Data were acquired from three independent experiments and presented as mean ± standard error mean (SEM).

### Silymarin showed an excellent binding affinity towards DENV-3 E protein

**Figure 7:**
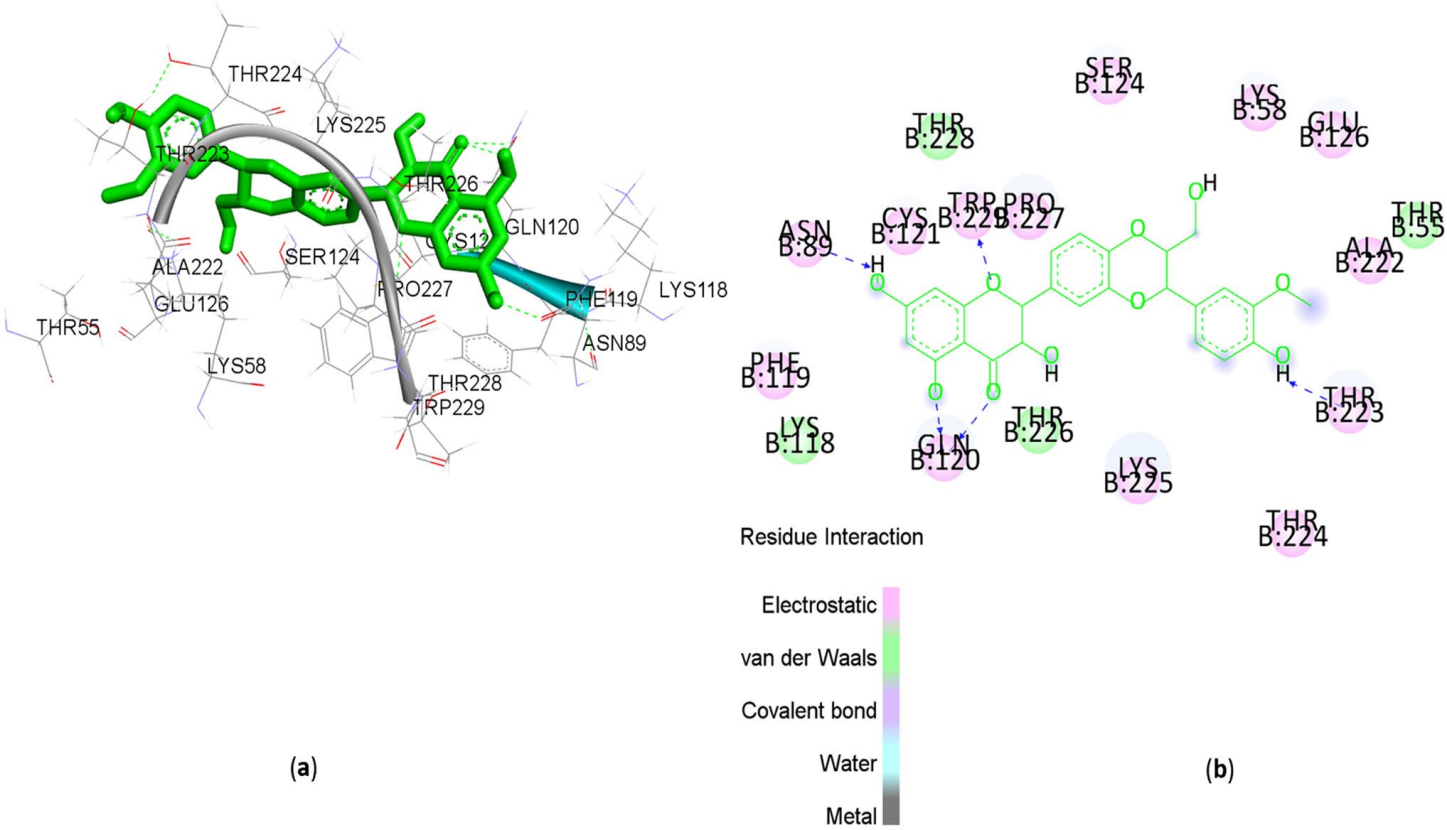
Interaction of silymarin with DENV E protein (1uzg). (**a**) 3D scheme of the silymarin interaction with DENV E protein (1uzg). (**b**) 2D scheme of silymarin interaction with DENV E protein (1uzg), including H-bonding and close contact. There are H-bonds at the (1uzg) E protein GLN120, TRP229, ASN89, and THR223.

Silymarin forms hydrogen bonding (H-bond) (Supplementary Table 1) and pi-cation interaction (Supplementary Table 2) with DENV E protein (1uzg). Supplementary Figure 1 showed the nine different conformations of silymarin with different E protein sites, which was ranked by AutoDock Vina. The strongest interaction affinity of silymarin was observed with the E protein at − 8.5 kcal/mol. Based on the strongest interaction, silymarin could form H-bonds with E protein at amino acid residues GLN120, TRP229, ASN89, and THR223 (Fig. 7). Besides that, silymarin binds to the E protein through close contact via PHE119, LYS118, THR226, LYS225, PRO227, THR228, SER124, THR224, LYS58, GLU126, ALA222, CYS121, and THR55 (Fig. 7).

## Discussion

Despite the global burden of dengue infection, there is no effective antiviral treatment, and the treatment still remains supportive. Thus, the need for antiviral drugs that have the potential to treat severe dengue infection is urgently needed. A vast amount of research has recently focused on screening for anti-dengue compounds such as small molecules^17^, nucleoside analogs^18^, antiviral peptides^7^, and natural plant extracts^19^.

Flavonoids are bioactive compounds found in plants that are less toxic and can be consumed in high amounts. Flavonoids such as quercetin have been reported to be a potential inhibitor, targeting the early stages of dengue infection in vitro^7^. Often, flavonoids such as baicalein and its derivative baicalin were reported to exhibit direct virucidal effects against DENV-2 sub-genotype New Guinea C (NGC)^14,15^. However, the anti-DENV potential of baicalein and baicalin has not been explored against other dengue serotypes. Silymarin extracted from milk thistle is another flavonoid reported to be a promising antiviral candidate against CHIKV^10^ and HCV^11^. Recently, silymarin and baicalein were reported to be potent antiviral agents against EV-A71^12^. Previous in silico studies showed silymarin can able to inhibit DENV NS4B with a binding affinity of more than − 8 kcal/mol^20^. However, not much is known about the antiviral properties of silymarin, baicalein, and baicalin against all four dengue serotypes. This study showed that silymarin and baicalein exhibited broad-spectrum antiviral activity against all four DENV serotypes.

Silymarin and baicalein were found to be well tolerated by Vero cells, but baicalin was less tolerated by Vero cells. Similar results were reported by Lalani et al.^12^, who evaluated the cytotoxicity effect of these flavonoids against EV-A71 in Rhabdomyosarcoma (RD) cells. However, in contradiction, Moghaddam et al.^15^ reported that the CC_50_ value of baicalin was at a much higher level of 290.9 µg/mL when tested in Vero cells. This may be due to the different sources of baicalin and in both studies. Based on the reduction in foci number, all three flavonoids did not show a prophylactic effect but show a promising virucidal effect against DENV-3.

Based on the reduction in foci number and viral RNA copy number, the SI values of silymarin, baicalein was higher than the SI of baicalin, and it can be concluded that silymarin and baicalein are more effective than baicalin in exerting virucidal effects against DENV-3. Also, silymarin and baicalein could inhibit 95.13% and 99.78% of DENV-3 infectivity at its highest non-toxic concentration compared to 76.75% of DENV-3 inhibition by baicalin. These findings were supported by two other studies by Moghaddam et al.^15^ and Zandi et al.^14^, who showed that baicalein has a lower IC_50_ value than baicalin, and thus, baicalein was more effective than its derivative when used against DENV-2.

Capability of infectious viral progeny able to spread to other parts of the body is one of the reasons for progression of mild to severe dengue. It is interesting to observe that baicalein and baicalin could inhibit the intracellular DENV-3 progeny infectivity, while silymarin require the virus to be released from the cells, thus inhibiting extracellularly. In addition, we investigated the effective time needed for these flavonoids to exert their virucidal effects against DENV-3. Our findings revealed that baicalein exhibited more effective virucidal activity against DENV-3 than silymarin and baicalin. Baicalein required only 15 min to reach up to 75% viral infectivity inhibition. In comparison, silymarin took 45 min of co-incubation with DENV-3 to achieve up to 75% viral infectivity inhibition, while baicalin could only achieve a maximum of 66.9% viral infectivity inhibition even after 60 min of contact with DENV-3. A previous study showed that silymarin is more effective and required a shorter period (< 1 min) of contact with EVA-71 to exert a 65% virucidal effect compared to baicalein which required a longer co-incubation time (60 min) to exert a maximum of 60% virucidal effect against EVA-71^12^.

The attachment assay revealed that baicalein exhibited lower viral infectivity, suggesting that it might bind the virus effectively compared to silymarin and baicalin. This might be due to the inability of baicalein-bound-virus to attach the surface of Vero cells. In the entry assay, silymarin was the most potential over the other two flavonoids to exhibit lower viral infectivity. It could bind to attached-DENV-3 to detach the virus from Vero cells' surface and prevent viral entry. Our findings suggest that the virucidal activity of flavonoids could be due to the binding of these compounds to the virus surfaces, thus preventing the viral attachment and entry.

Various compounds have been shown to target DENV structural proteins and inhibit DENV infectivity directly. These molecules might bind to the viral envelope or capsid proteins and thus reduce viral infectivity by inhibiting viral attachment or preventing viral entry or uncoating in the host cells. For example, 2,4-disubstituted pyrimidines (2-12-2 and 7-148-6) are small molecules proven to have antiviral properties by binding to the β-OG pocket of the DENV E protein and thus inhibiting DENV entry^21^. Another compound, ST-148, was reported to enhance capsid protein self-interaction, induced structural rigidity, and disturbed the assembly of DENV nucleocapsids ^22^. Our findings showed that silymarin, baicalein, and baicalin could bind to DENV-3 and reduce viral infectivity, possibly interacting with DENV structural proteins. These interactions between flavonoids and DENV structural proteins might block DENV attachment to its cellular receptors such as heparan sulfate, nLc4Cer, DC-SIGN, mannose receptor, HSP70/HSP90, and GRP78^23^.

Our study is the first to report the virucidal activity of silymarin against extracellular DENV particles, which could be due to the interaction of silymarin with DENV E protein. Molecular docking was employed to further study the in silico mechanism of action of silymarin against DENV E protein. E protein of DENV is known to facilitate viral attachment and entry into the host cells. It also plays a significant role during the fusion stage, where it triggers the fusion of viral particles to the endosomal membrane, leading to the release of the viral genome into the host cells. Our in silico findings showed that most of the binding interactions between silymarin and E protein could occur at domain 2 of the E protein. Domain 2 of E protein is an elongated domain containing a fusion loop that is conserved among *Flavivirus*. The docking study showed strong H-bonds between silymarin and the E protein of the dengue virus, with the highest binding affinity of − 8.5 kcal/mol. The lengths of H-bonds range from 1.4 to 2.4 Å, and Pi-cation interactions were also observed between silymarin and the E protein, making the binding more robust and stable. Based on the model with the best binding interaction, silymarin can form strong H-bonds with amino acid residues GLN120, TRP229, ASN89, and THR223 of the DENV E protein. Among these residues, ASN89 residue was reported to participate in stabilizing the fusion loop of DENV E protein during the fusion stage^24^. Our in-silico and in vitro findings were consistent, proving that silymarin could exert neutralizing properties against extracellular DENV by inhibiting the attachment of DENV to the host cells.

In conclusion, our findings revealed that silymarin could reduce the DENV infectivity through direct interactions with viral surface proteins, thus preventing viral attachment and entry. Baicalein could inactivate both the extracellular DENV-3 and the newly released viral progeny. Since baicalin was more toxic to the Vero cells with limited antiviral activity against DENV-3, we did not further investigate this compound. The broad-spectrum virucidal activity of silymarin and baicalein against all four DENV serotypes raising the possibility of employing both antiviral agents synergistically. The molecular mechanism of how these flavonoids target DENV and the potential resistance of DENV towards these flavonoids need to be further explored. Outcome of this study warrant further evaluation of antiviral activity of these flavonoids in vivo and provide insights for future development of effective antiviral agent against all four dengue serotypes.

## Methods

### Cell and viruses

DENV-1 (Hawaii), DENV-2 (New Guinea C), DENV-3 (H87), DENV-4 (H241), and Vero (African green monkey kidney, ATCC○R CCL-81TM) cell line were purchased from American Type Culture Collection (ATCC, USA). Vero cells were used for virus propagation, titration and the evaluation of the antiviral activity of flavonoids. Cells were cultured in DMEM (Gibco, USA) supplemented with 10% Foetal Bovine Serum (FBS) (Gibco, USA) and 1% penicillin–streptomycin antibiotics (PSA) (Gibco, USA) and maintained at 37 °C in 5% CO_2_. Four dengue serotypes were propagated in Vero cells with 2% FBS for 4–10 days. The collected viral supernatant was then clarified by centrifugation 3100*g*, aliquoted in 1.5 ml tubes supplemented with 20% FBS, and stored at − 80 °C. Viral titre was quantified using a modified plaque assay.

### Foci Forming Unit Assay (FFUA)

The viral titres of all four dengue serotypes were quantified using FFUA according to a previous study^25^. The Vero cells (1 × 10^5^ cells/well) were seeded overnight in 24 well-plate. Monolayer confluent Vero cells were infected with ten-fold serial diluted viral stock (200 μL/well) for 1 h in 37 °C. After one hour of infection, unabsorbed virus suspension was discarded, and the cells were washed with PBS. Then DENV infected Vero cells were overlaid with immobilizing media, consisting of medium viscosity carboxymethyl cellulose (CMC) (Sigma, Japan) and DMEM supplemented with 2% FBS, and further incubated for 4 days at 37 °C to allow the formation of countable foci. After 4 days of incubation, CMC was removed, and cells were fixed with acetone: methanol (Merck, Germany) (1:1) fixing solution for 1 h. After that, the fixing solution was discarded, and the cells were washed with PBS. After washing, the primary antibody (4G2), harvested from HB112 hybridoma cells (ATCC, USA), diluted in 5% skim milk (blocking buffer) (Thermo Fisher, UK) was added and incubated for 1 h at 37 °C. After that, the primary antibody was washed with PBS, and HRP-conjugated secondary antibody (Invitrogen, USA) was added at a ratio of 1:1000 diluted in blocking buffer and further incubated for 1 h at 37 °C. After 1 h of incubation, the secondary antibody was washed away, and TrueBlue peroxidase substrate (Seracare, USA) was added into each well in the dark. Foci in each well were counted, and viral titer was determined in foci forming unit per microliter (FFU/mL).

### Flavonoid compounds

Silymarin, baicalein, and baicalin were purchased from Sigma-Aldrich (St. Louis, USA). These flavonoids were dissolved in DMSO (ATCC, USA) to yield 50 mg/mL stocks and kept at − 80 °C until use. The flavonoid stocks were further diluted in 2% FBS-DMEM to prepare the working stocks and to minimize the DMSO concentration in the assays.

### Cytotoxicity assay

The cytotoxic potential of flavonoids in Vero cells was assessed by MTS assay. Briefly, confluent Vero cells in a 96 well plate were treated with different concentrations of flavonoids in triplicates with DMEM containing 2% FBS. After 2 days, 20 μL of 3-(4,5-Dimethylthiazol-2-yl)-5-(3-carboxymethoxyphenyl)-2-(4-sulfophenyl)-2H-tetrazolium (MTS) reagent (Promega, France) was added to each well. After 1 h of incubation at 37 °C, the cells were agitated, and the absorbance was measured at 490 nm in a microplate reader (Tecan, Infinite M2000, Switzerland). Maximum non-toxic dose (MNTD_80_) and 50% cytotoxic concentration (CC_50_) were calculated by non-linear regression using prism software (GraphPadPrism8, USA).

### Focus Forming Unit Reduction Assay (FFURA)

The antiviral activity of flavonoids was determined by measuring the reduction in the number of DENV infectious foci after treatment. The antiviral assays were performed based on Zandi et al.^14^ with minor modifications. Briefly, a confluent monolayer of Vero cells seeded in 24-well plates was treated with flavonoids before or after or with the virus infection and incubated for 4 days. The virus foci were visualized using immunostaining. The flavonoids' antiviral activities were determined by calculating the percentage of foci reduction (%RF) according to the following equation:

$${\text{Percentage}}\;{\text{of}}\;{\text{inhibition}}\left( \% \right) = \frac{{(\varvec{C} - \varvec{T})}}{\varvec{C}} \times 100\%$$

where, ***C*** is the number of foci without the flavonoid treatment (control). ***T*** is the number of foci with the respective flavonoid treatment.

The percentage of reduction in virus particles was represented as the mean ± standard error of the mean (SEM) from triplicate assays determined from three independent experiments.

### Viral RNA extraction and quantitative real-time polymerase chain reaction (qRT-PCR)

Viral RNA extraction was performed using QIAamp^R^ Viral RNA extraction kit (QIAGEN, Germany), and viral RNA copy number was quantified using the genesig Real-Time qRT-PCR Standard Kit (PrimerDesign, UK). The reactions were in a final volume of 20 μL containing 10 μL of the master mix, 1 μL of probe/primer mix, 4 μL of nuclease-free water, and 5 μL of viral RNA template. Quantitative PCR was performed using a CFX96 Touch™ Real-Time PCR Detection System (Bio-Rad, USA.) with the following conditions: 10 min at 55 °C (reverse transcription), 2 min at 95 °C (enzyme activation) followed by 50 cycles of amplification (10 s at 95 °C, 60 s at 60 °C). The viral RNA copy number was determined based on the threshold cycles (Ct) and standard curve generated from known RNA standards.

### Cell protection assay

Cell protection assay was performed to determine the prophylactic effect of flavonoids against DENV-3, Vero cells were first treated with 200 µg/mL of silymarin, 100 µg/mL of baicalein and 20 µg/mL of baicalin and incubated at 37 °C. After 1 h, the compounds were removed, and the cells were infected with DENV-3 (80 FFU/well) and incubated at 37 °C for virus absorption. After 1 h, the unabsorbed virus was removed, and the cells were overlaid with 1 mL of overlay medium. After 4 days, the reduction in foci was determined by immunostaining.

### Virucidal assay

To determine the viral-inactivation effects of flavonoids against DENV, DENV-1, 2, 3 and 4 (80 FFU/well) were first treated with serially diluted flavonoids: up to 200 µg/mL of silymarin, 100 µg/mL of baicalein, and 20 µg/mL of baicalin. After 1 h, the flavonoid-treated virus was used to infect Vero cells at 37 °C for 1 h. Then, the unabsorbed virus was removed, and the cells were washed and overlaid with 1 mL of overlay medium. After 4 days, the reduction in foci was determined by immunostaining. Another parallel experiment was carried out by replacing the overlay medium with DMEM supplemented with 2% FBS to determine the reduction in viral RNA copy number by qRT-PCR as previously described.

To determine the activity of flavonoids in inhibiting the intracellular viral progeny, Vero cells (1 × 10^5^ cells/well) in a 24-wells plate were infected with DENV-3 (MOI = 0.1) for 1 h at 37 °C. Then, the unabsorbed virus was removed, and the cells were washed and treated with serially diluted flavonoids: 200 µg/mL of silymarin, 100 µg/mL of baicalein, and 20 µg/mL of baicalin for 48 h at 37 °C. After 48 h, the viral supernatant was collected, and twofold dilution was used to reinfect fresh monolayer of Vero cells, and the reduction in viral titer was quantified using FFURA and qRT-PCR.

### Time course assays for virucidal activity

Vero cells (1 × 10^5^ cells/well) were grown overnight in a 24-well plate. DENV-3 (80 FFU/well) suspension was treated with 200 µg/mL silymarin, 100 µg/mL of baicalein and 20 µg/mL of baicalin for 0, 5, 15, 30, 45 and 60 min. Cells were infected with the flavonoid-treated virus and incubated at 37 °C for 1 h. Then, the unabsorbed virus was removed, and the cells were washed and overlaid with 1 mL of overlay medium. After 4 days, the reduction in foci was determined by immunostaining.

### Attachment assay

Vero cells (1 × 10^5^ cells/well) were grown overnight in a 24-well plate. DENV-3 (80 FFU/well) suspension was treated with 200 µg/mL silymarin, 100 µg/mL of baicalein, and 20 µg/mL of baicalin at 37 °C. After 1 h, the flavonoid-treated virus is incubated on ice for 15 min. Next, pre-chilled Vero cells were infected with the pre-chilled flavonoid-treated virus at 4 °C for 1 h to allow virus attachment. Then, the unabsorbed virus was removed, and the cells were washed and overlaid with 1 mL of overlay medium. After 4 days, the reduction in foci was determined by immunostaining.

### Entry assay

Pre-chilled DENV-3 (80 FFU/well) in the absence of flavonoids were added to the pre-chilled Vero cells and incubated at 4 °C to allow virus attachment. After 1 h, the unattached virus was removed, and the cells were washed and treated with pre-chilled flavonoids: 200 µg/mL silymarin, 100 µg/mL of baicalein, and 20 µg/mL of baicalin at 37 °C for 1 h to allow virus entry. After that, the medium containing flavonoids was removed, and Vero cells were washed and treated with acidic PBS (pH 3) for 60 s to inactivate the extracellular virus. After 60 s, the acidic PBS was neutralized by alkaline PBS (pH 11) in each well. Next, cells were then washed with serum-free media and overlaid with 1 mL of overlay medium. After 4 days, the reduction in foci was determined by immunostaining.

### Fluorescence-activated cell sorting (FACS) neutralization test (FNT)

Vero cells were seeded in 24 well plates at a density of 1 × 10^5^ cells/well and incubated overnight. The antiviral assays were done using MOI 0.5 DENV-3, and FNT was carried out 48 h post-infection. Viral infected cells were harvested by trypsinization and centrifugation (1500 rpm, 5 min). Harvested cells were washed with PBS and fixed with 250 µL of BD Cytofix/Cytoperm™ Fixation/Permeabilization solution kit with BD GolgiStop™ (BD Biosciences, USA) solution and incubated on ice for 20 min in the dark. The cells were centrifuged at 3000 rpm for 2 min and washed two times with stain buffer (PBS + 5%FBS). Staining was performed with 50 µL of anti-dengue virus type III primary antibody, clone 5D4-11 (MAB8703 Sigma-Aldrich, USA), diluted in Cytoperm/Cytowash solution at a ratio of 1:1000 and incubated for 1 h on ice. After that, the cells were washed and stained with 50 µL of FITC-conjugated secondary antibody (Sigma-Aldrich, USA) diluted in Cytoperm/Cytowash solution at a ratio of 1:1000 and incubated for 1 h on ice in the dark. The samples were analyzed in a BD FACSCalibur flow cytometer (BD Biosciences, USA) using BD Cellquest pro software. For each sample, 10,000 events were collected, and the percent reduction in the number of infected cells was calculated using the following equation.

$${\text{Percentage}}\;{\text{of}}\;{\text{inhibition}}\left( \% \right) = \frac{{({\varvec{C}} - {\varvec{T}})}}{\varvec{C}} \times 100\%$$

where,*** C*** is the percentage of viral infected cells among the 10,000 events without flavonoid treatment (control). ***T*** is the percentage of infected cells among the 10,000 events with the respective flavonoid treatment.

Percent reduction in virus-infected cells was represented as the mean ± standard error of the mean (SEM) from triplicate assays determined from three independent experiments.

### In silico molecular docking

The three-dimensional structure of DENV-3 E protein (1uzg) was downloaded from the Protein Data Bank (PDB) (https://www.rcsb.org/). Using Discovery studio 3.5 software (https://discover.3ds.com/discovery-studio-visualizer-download), E protein structure was minimized by applying CHARMM27 force field. All the water molecules and ligands were removed from the E protein before the docking process. The silymarin structure was downloaded from PubChem (https://pubchem.ncbi.nlm.nih.gov/) and then imported into Discovery studio 3.5 software to be minimized before the docking process. Autodock Vina 1.5.6 (http://vina.scripps.edu/) was used to add hydrogen molecule into both E protein and silymarin for running and to save both structures as PDBQT files. Before molecular docking analysis, E protein was fit into a grid box, and the grid information was noted in a text file. The grid that was used to run E proteins was center-X = 13, center-Y = − 2.719, center-Z = 15, size-X = 64, size-Y = 40, size-Z = 126. In this study, blind molecular docking was carried out using Autodock Vina 1.5.6 software to examine silymarin and E protein interactions. AutoDock Vina ranked different conformations of silymarin with each E protein based on their binding affinity energy. PyMoL software was used to make all conformations into a single file before visualizing in Discovery Studio 3.5. Discovery Studio 3.5 gave data on close contact, hydrogen bonding, pi–pi interaction, and pi–cation interaction between silymarin and E protein.

### Data and statistical analysis

The maximum non-toxic dose (MNTD_80_) and 50% of inhibition (IC_50_) of flavonoids in Vero cells were calculated using Prism software (GraphPadPrism8, CA, USA) by applying four-parameter logistic non-linear regression model. The selectivity index (SI) was calculated as SI = CC_50_/IC_50_. Data presented are the mean ± SEM of three independent experiments. The student t-test was used to analyze the antiviral activity of flavonoids compared to a positive control (*p < 0.05, **p < 0.01, ***p < 0.001, ****p < 0.0001).

## Supplementary Information

Supplementary Information.

## Data Availability

The authors confirm that the data supporting the findings of this study are available within the article and in the supplementary information file.
