# Supplementary material for: Antiviral activity of silymarin and baicalein against dengue virus
Source: Sci Rep. 2021 Oct 27;11:21221. doi: 10.1038/s41598-021-98949-y (PMC8551334; doi:10.1038/s41598-021-98949-y)
Supplement: Supplementary file 1 — Supplementary Information. [file 41598_2021_98949_MOESM1_ESM.pdf]

Supplementary Table 1. Hydrogen bonding of silymarin with DENV E protein (1uzg). This table shows the H-bond interaction of silymarin with different E protein residues at different affinities ranked by AutoDock Vina 1.5.6. The third column shows the length of each H-bond between the molecule and residue. The last column shows the affinity energy, which was ranked by AutoDock Vina 1.5.6.

Complex	Interaction	Distance (Å)	Affinity (Kcal/mol)
conf.1+E	B:GLN120:HE21 - :COMPOUND:O6	2.19556	-8.5
	B:GLN120:HE21 - :COMPOUND:O7	2.07167	-8.5
	B:TRP229:HE1 - :COMPOUND:O3	2.43571	-8.5
	:COMPOUND:H53 - B:ASN89:OD1	2.21109	-8.5
	:COMPOUND:H54 - B:THR223:OG1	2.20495	-8.5
conf.2+E	A:GLN120:HE21 - :COMPOUND:O6	1.96793	-8.1
	A:GLN120:HE21 - :COMPOUND:O7	1.91219	-8.1
	A:LYS225:HZ2 - :COMPOUND:O4	1.74175	-8.1
	A:TRP229:HE1 - :COMPOUND:O3	2.36725	-8.1
	:COMPOUND:H51 - A:SER124:OG	1.65787	-8.1
conf.3+E	B:GLN120:HE21 - :COMPOUND:O6	2.36058	-8.1
	B:GLN120:HE21 - :COMPOUND:O7	1.86173	-8.1
	:COMPOUND:H53 - B:ASN89:OD1	2.41898	-8.1
conf.4+E	A:GLN120:HE21 - :COMPOUND:O6	1.89507	-7.9
	A:GLN120:HE21 - :COMPOUND:O7	2.23612	-7.9
	A:SER124:HG - :COMPOUND:O2	2.44181	-7.9
	A:LYS225:HZ2 - :COMPOUND:O4	1.73866	-7.9
	:COMPOUND:H53 - A:PRO227:O	2.18388	-7.9
conf.5+E	:COMPOUND:H52 - A:GLU154:OE1	1.88805	-7.8
	:COMPOUND:H53 - B:ALA243:O	2.17225	-7.8
	:COMPOUND:H54 - B:GLN246:O	2.11572	-7.8
conf.7+E	A:LYS110:HZ3 - :COMPOUND:O10	1.98159	-7.5
	B:ASN8:HN - :COMPOUND:O8	1.88598	-7.5
conf.8+E	B:SER124:HG - :COMPOUND:O2	2.37509	-7.5
	B:LYS225:HN - :COMPOUND:O8	1.91505	-7.5
	B:LYS225:HN - :COMPOUND:O10	2.4792	-7.5
	:COMPOUND:H51 - B:CYS121:O	2.24968	-7.5
conf.9+E	A:THR263:HG1 - :COMPOUND:O10	2.24769	-7.4
	:COMPOUND:H53 - A:ILE276:O	2.40464	-7.4

Supplementary Table 2. Pi interaction between silymarin and DENV E proteins. This table shows the pi-cation interactions of silymarin with DENV E protein (1uzg). The distance of interaction is shown in the third column.

Proteins	Binding Interaction	Distance (Å)
E protein	:COMPOUND - A:LYS225:NZ	5.25161
	:COMPOUND - B:ARG99:NE	5.79745
	:COMPOUND - B:LYS244:NZ	3.59212
	:COMPOUND - B:LYS58:NZ	4.28452
	:COMPOUND - B:LYS225:NZ	6.8811
	:COMPOUND - A:LYS225:NZ	5.44641
	:COMPOUND - B:LYS225:NZ	5.82817

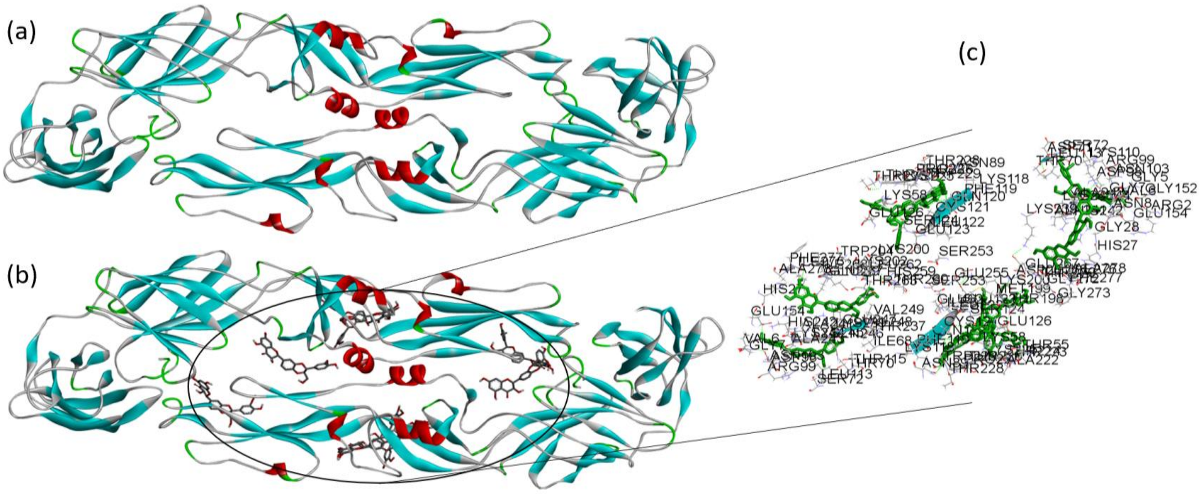

Supplementary Figure 1. DENV E protein (1uzg) vs. silymarin. Figure 8(a) DENV E (1uzg) protein structure. Figure 8(b) DENV E (1uzg) protein with silymarin with nine different possible binding sites. Figure 8(c) Close contact and interaction residues between DENV E (1uzg) protein with silymarin with nine different possible binding sites.

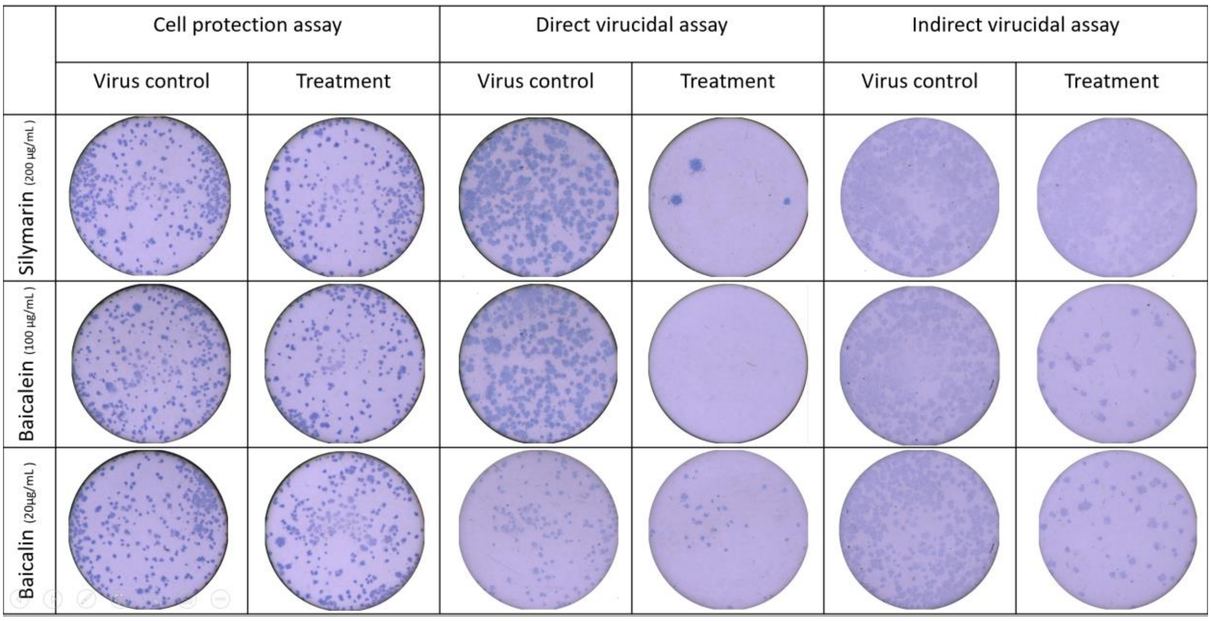

Supplementary Figure 2. Antiviral Activity of Silymarin, Baicalein, and Baicalin against DENV-3. The foci reduction by silymarin (200 µg/mL), baicalein (100 µg/mL), and baicalin (20 µg/mL) at different time point: cell protection or pre-infection stage, direct virucidal and indirect virucidal stage.
